# Pneumatization Pattern and Status of the Mastoid Antrum in Chronic Otitis Media: A Review

**DOI:** 10.7759/cureus.27068

**Published:** 2022-07-20

**Authors:** Farhat Q Khan, Prasad T Deshmukh, Sagar S Gaurkar

**Affiliations:** 1 Otolaryngology - Head and Neck Surgery, Jawaharlal Nehru Medical College, Wardha, IND; 2 Otolaryngology - Head and Neck Surgery and Surgical Oncology, Jawaharlal Nehru Medical College, Wardha, IND

**Keywords:** temporal bone pneumatisation, diploic mastoid, chronic otitis media, mastoid antrum, pneumatisation pattern

## Abstract

Chronic otitis media is generally more prevalent in individuals with a weakly pneumatized temporal bone, whereas acute otitis media has a propensity for pneumatized temporal bone. Antimicrobial drugs are thought to have influenced the incidence and progression of middle ear infections. The mastoid air cell system, a part of the middle ear cleft, has recently been recognized as a crucial factor in the genesis, behaviour, course, and outcome of middle ear inflammatory conditions. Epithelium infiltrates the growing bone and produces epithelium-lined air cell chambers, a process known as pneumatization.

Conventional temporal bone radiography, i.e. X-ray mastoid, has not kept up with recent otology breakthroughs. Detailed visualization of the aural structures has advanced significantly with the introduction of high-resolution computed tomography (HRCT). HRCT has a clear edge in the assessment of the temporal bone, especially when thin-section, high-resolution methods are used, resulting in a more precise description of the pneumatization pattern and the anatomical extent of middle ear pathology.

Our results of the review indicated that persistent inflammation of the middle ear in children inhibits pneumatization of the temporal bone. Due to its potential to exert a greater negative middle ear pressure, the middle ear volume is insufficient to generate a retraction pocket; thus, the size and state of the antrum is also a significant factor in the establishment of a COM-like retraction pocket in inactive squamosal disease. Numerous factors, including the number of patients, regional, genetic, ethnic and hereditary characteristics, as well as the cellularity of the mastoid, which is impacted by a multitude of factors, may account for the variable reports and inter-study variation in this regard.

## Introduction and background

Otitis media (OM) is an inflammation of the cleft of the middle ear, that is composed of the tympanic cavity, aditus, antrum, mastoid air cell system, and eustachian tube [1]. Chronic suppurative otitis media (CSOM), now referred to as chronic otitis media (COM), is a chronic inflammation of the middle ear that also includes, to a greater or lesser extent, concomitant inflammation of the mastoid air cell system, due to its anatomical proximity to the middle ear [2]. The distinctive presentation is chronic or persistent otorrhoea over two to six weeks through a ruptured tympanic membrane [3].

As per "the environmental theory", which emphasizes that the degree of mastoid pneumatization is governed by environmental indices, middle ear infections in children may lead to inflammatory alterations in the middle ear mucosa and the development of temporal bone [4]. According to the genetic hypothesis, the extent of mastoid pneumatization is genetically determined [5]. The debate over whether the underdeveloped mastoid is an inherited characteristic or the result of acute otitis media in childhood has raged for years [6].

The mastoid air cell system, which is part of the middle ear cleft, has recently been identified as a significant component in the genesis, behaviour, course, and outcome of middle ear inflammatory diseases. Pneumatization is the process in which epithelium infiltrates the developing bone and creates epithelium-lined air cell cavities.

Chronic otitis media was more frequent in those with a poorly pneumatized temporal bone, whereas acute otitis media was more prevalent among people with a completely pneumatized temporal bone. Antimicrobial agents are claimed to have altered the incidence and progression of otitis media [4].

The main objectives of this review were: (1) to provide a reference for the correlation of the mastoid air cell pneumatization so as to address a variety of dimensions of chronic otitis media; and, (2) to find the correlation of status of the mastoid antrum and pneumatization pattern in various chronic otitis media.

## Review

Both as a reservoir and an active cavity, the mastoid air cell system may exchange gas independently of the eustachian tube. Within the air cell system, the submucosal capillary network enables gas exchange. Due to the fact that gas exchange occurs in the cellular mucosa, the total mucosal surface area affects the rate of gas exchange. It has been demonstrated that the mastoid air cell system works as an air reservoir. By supplying air to the middle ear, the mastoid cavity offsets the effects of pressure changes in the middle ear. This system's capacity is its volume [7].

The evolution of air-filled spaces inside the bone is known as pneumatization. Pneumatization mostly occurs through the eustachian tube to the middle ear and the mastoid process of the temporal bone. The creation of air cells is attributed to the activity of the sub-epithelial layer, according to Whittmack [8]. Sound reception, resonance, insulation, acoustic dissipation, air reservoir activity, protection from external stress, and decreasing the weight of the skull are all functions of the temporal air cells. Pneumatization of the temporal bone generally follows the Gaussian pattern of distribution in people.

Both the bone marrow and dense bone in diploic and sclerotic mastoids lack pneumatization. It is believed that pneumatized cells grow between the 22nd and 24th weeks of embryonic development. Mastoid cell pneumatization occurs in the 33rd week of gestation and lasts until the age of eight or nine. Around the 35th week, the antrum (largest air cell) achieves adult size. The synthesis of air cells begins with the formation of bone cavities. The physiological process involved in this development is dependent on periosteal activity. The primordial bone marrow in the bone cavities evolves into mesenchymal connective tissue. After invagination, the epithelial mucous membrane atrophies, leaving a thin remnant membrane attached to the periosteum. The number of air cells increases when the membrane recedes and the subepithelial bone is reabsorbed [7,9].

The physiology of the middle ear is greatly influenced by the mastoid air cell system. The mastoid air cells, according to Tumarkin and Holmquist et al., serve as an air reservoir for the middle ear and aid in the regulation of middle ear pressure. Sadé was in favour of the notion [6,10-12].

The interaction between blood vessels and the mucosal basement membrane is closer in the mastoid air cells than in other regions of the middle ear. According to Okubo, the human mastoid cell's histological structure is very comparable to that of the lung and nasal membranes, which are responsible for gas effusion and diffusion [13,14].

Affecting factors

Until adolescence, the mastoid air cell system is typically bigger in females. This is likely a result of the female species' generally faster physical development. Nevertheless, there is likely no substantial gender difference in adult aerated mastoid size [15]. Populations of diverse ethnic origins exhibit a similar development trend, however, there may be a size disparity that follows body size. This is supported by studies of paediatric populations in Scandinavia, India, Japan, and Africa [16,17].

The research indicates that the degree of pneumatization of the temporal bone may be related to the development of middle ear infection and cholesteatoma. However, it is uncertain if inadequate pneumatization is the cause or effect of middle ear infection. The pneumatization of the temporal bone is separated into five compartments: the middle ear, the squamomastoid (mastoid), the perilabrynthine, the petrous apex, and the accessory. There are squamous, zygomatic, occipital, and styloid cells in accessory areas [13].

The pneumatization of the mastoid area is classified as [9]: (1) Cellular mastoid (complete pneumatization); (2) Diploic mastoid (partial pneumatization); (3) Sclerotic mastoid (absent pneumatization).

Mastoid pneumatization and middle ear disorders remain a hot topic of debate. Table 1 depicts various hypotheses on this fact in the current body of research.

**Table 1:** Various theories and hypotheses of pneumatization of the mastoid

Theory	Hypothesis/Ideas proposed
Tumarkin’s theory [11]	Eustachian tube obstruction with resultant intratympanic vacuum leads to the arrest in pneumatization. Usually occurs in children as a result of infection and enlargement of adenoids.
Diamant's hereditary (genetic, normal variant) hypothesis [18]	The degree of pneumatization is determined genetically. Impaired pneumatization is a risk factor for otitis media, either acute or chronic. He estimated that the average size of his patients' cellular systems was 12 cm^2^.
Environment hypothesis [5]	Tos's tympanometric exams of healthy children from infancy to school age children with normal tympanometric scores had a bigger air cell system than children with abnormal tympanometric scores. Besides, he found that the length of the secretory otitis media had caused the air cell system to shrink.
Wittmaack’s endodermal hypothesis [8]	Healthy middle ear mucosa is necessary for proper pneumatization, which might be hampered by inflammation or tubal dysfunction. Therefore, middle ear illnesses are responsible for newborns' and children's delayed pneumatization.
Graham and Brackmen’s theory [9]	The size of the mastoid depends upon the final size of the skull in an individual as in acromegaly (large mastoid) and microcephaly (underdeveloped mastoid).

Pneumatization as studied by various authors

The relationship between the size of the mastoid air cell system and the progress of the middle ear cavity disease was first addressed by Flisberg [19]. Later, Holmquist and Bergstrom suggested that the extent of mastoid pneumatization influences the efficacy of middle ear surgery. Tympanoplasty with mastoidectomy results in much more middle ear retraction after surgery than tympanoplasty without mastoidectomy [12]. The failure of tympanoplasty in children may be attributed to an undeveloped air cell system, according to Bonding [20].

Roy et al. discovered that 1.92% of mastoids were pneumatized, 19.23% were diploic, and 78.85% were sclerotic. Contralateral normal mastoids were pneumatized in 42.86% of cases, diploic in 31.43%, and sclerotic in 25.71% of instances [21]. Sadé observed an 82.3% association between a low or non-pneumatized mastoid and squamosal COM, whereas Gomaa et al. reported a 60.7% association [14,22-24].

Numerous additional researchers have associated the pattern of pneumatization of the temporal bone on HRCT with COM as shown in Table 2.

**Table 2:** Comparative depiction of pneumatization in patients of chronic otitis media.

AUTHORS	PNEUMATIC	DIPLOIC	SCLEROTIC
Rai et al. [2]	44%	6%	50%
Sunitha et al. [25]	33.3%	3.7%	53.7%
Kanotra et al. [26]	0%	0%	100%
Datta et al. [27]	4%	0%	96%
Our ongoing study	21.6%	28.3%	50%

Imaging and volume of mastoid pneumatization:

Traditional temporal bone radiography, i.e. X-ray mastoid, has not kept pace with advances in modern otology, and as a result, many otologists believe temporal bone radiography to be of limited use in COM. Many surgeons believe that, with expertise and meticulous procedures, the breadth and character of the disease may be established during surgical exploration without the need for prior information.

With the introduction of HRCT, however, imaging of the ear's structures has made significant improvements. HRCT has had a significant impact on the surgical management of middle ear diseases. It validates and elucidates otoscopic findings, resolves clinical difficulties, and, in many cases, aids the decision-making process when surgery is necessary. HRCT outcomes can be used to plan the surgical technique.

Imaging with a computed tomography (CT) scan permits a full preoperative study of the anatomical variances and bone features of the middle ear, as well as the ossicular chain and soft tissue. HRCT has clearly demonstrated its advantage in the examination of the temporal bone, especially using thin-section, high-resolution methods, therefore providing a more exact characterization of the pneumatization pattern of the bone and the anatomical extent of middle ear illness [28].

Around 72-99% of the common population exhibits symmetrical pneumatization. When asymmetric pneumatization is apparent on CT, the ear with the depressed system should be suspected of having a disease. For new bone growth and destruction, the shape of air cells must be carefully examined.

When analyzing the mucosal thickness, locations with partial pneumatization might be mistaken as sites of infection. High-resolution computed tomography can be utilized to assess pneumatization of the temporal bone since this technique has the benefit of displaying pneumatization in its entirety with high resolution.

According to Todd, computed tomography is the optimal imaging technique for assessing mastoid pneumatization. The mean value of mastoid pneumatization on 30 adult cadaver specimens was determined by Todd to be 7.59±3.9 ml. Sadé noticed a mastoid pneumatization volume of 12.9±4 cm^2^ in 150 normal ears and 17.4±5 cm^2^ in 150 osteosclerotic ears [6,29].

The results of mastoid measures among people of varied origins were comparable (Scandinavian population, Indians, Americans, and Israelis). According to a recent survey that used 3D CT reconstruction to assess the volume of the mastoid air cells, the adult mastoid loses volume with age. However, because the study was conducted as a cross-sectional observation rather than a longitudinal cohort, interpreting an older, smaller mastoid as shrinking may be questionable, i.e., comparing different generations with different histories of nutrition and health conditions that may have influenced the mastoid and growth of total body size. It is noteworthy that a comparison between the size of mastoid air cells measured before and after the onset of symptoms and the intensive use of antibiotics did not reveal a difference [15,17]. Similarly, Ueda reported such a finding [16].

Antrum and mastoid pneumatization

The cross-sectional study was conducted by the authors at Acharya Vinoba Bhave Rural Hospital (AVBRH), Wardha, between 2019 to 2022, among 60 patients of COM belonging to both genders between the age group of 15-65 years, to analyze the status of the mastoid antrum and pneumatization pattern, showed the mastoid antrum was normal in two (3.3%) patients. Oedematous mucosa in six (10%), granulations in the antrum in almost two-thirds of the patients (43,71.6%), and cholesteatoma in nine (15%) patients were other important features observed in the mastoid antrum. Of the 43 patients who had granulations in the antrum, 11 (18.3%) patients presented with central perforation while 13 patients (21.6%) presented with subtotal perforation. A large number i.e., 19 patients (31.6%) with granulations had posterosuperior retraction pocket (PSRP). Cholesteatoma in the antrum on exploration was observed in nine (15%) and all of them clinically presented with PSRP. As compared to large and subtotal perforation, granulations in antrum were seen more frequently in PSRP. This was shown to be a statistically significant difference (p-value=0.011). Statistical analysis was conducted with descriptive and inferential statistics using the chi-square test; the software programmes used in the analysis were SPSS 27.0 version (IBM Corp., Armonk, NY) and GraphPad Prism 7.0 version (GraphPad Software, San Diego, CA) and p<0.05 is considered to be significant.

Depth of mastoid antrum was measured in all the 60 patients intraoperatively using the depth measuring probe of a modified Vernier calliper, superiorly extending from the outer bony cortex over the Mac Evan’s triangle till the bulge of lateral semicircular canal inferiorly. The total mean depth was found to be 17.27 mm. The average depth of antrum was almost similar in patients with large central perforation and subtotal perforation taken together (16.75 mm) and in patients with PSRP (17.75 mm). The maximum number of patients had cholesteatoma (eight out of nine), the depth was more than 18.10 mm while the majority of patients with granulation tissue (22,36.6%) had a depth between 15.10 -18.00 mm.

In our study, intraoperatively granulation tissue was seen in 43 patients out of (71.6%). On its correlation with mastoid pneumatization, it was found that 35 out of 43 (81.39%) had either diploic or sclerotic mastoid as against 8 (18.6%) in which cellular mastoid was observed. Similarly, all the patients in whom cholesteatoma was found in the antrum had either sclerotic or diploic mastoid. This was statistically found to be significant (p=0.026).

Grewal et al. found commonly a big antrum in ears with retraction pockets. Around 76% of the 42 individuals whose antrums were inspected after surgery had a big antrum, whereas 24% had a tiny, confined antrum, according to their study. The presence of a big antrum in an atelectatic ear casts doubt on the commonly held idea that an atelectatic ear possesses weakly pneumatic or sclerotic mastoid air cells [30].

In 1918, Wittmaack hypothesized that infantile sterile otitis media neonatorum or non-secretory otitis media occurring shortly after birth may cause persistent residual fibrosis and subsequent epithelial tissue thickening. This would cause many adhesions in the middle ear, disrupting the normal pneumatization process. Such an "apneumatic mastoid" would make the ear susceptible to atelectasis [8]. However, the presence of bone-eroding disease in the antrum, such as cholesteatoma or granulations as observed by Kobayashi et al. [30] may be a contributory factor. Table 3 is a comparative depiction of various authors and their intraoperative findings on the status of the mastoid antrum.

**Table 3:** Comparative depiction of intraoperative findings of the status of mastoid antrum by various authors

Authors	Status of mastoid antrum		
	Granulation	Cholesteatoma	Others (normal antrum, oedema, cholesteatoma with granulations, polyp)
Solanki et al. [31]	55.07%	21.73%	23.18 %
Azevedo et al. [32]	63%	21%	16%
Sharma et al. [33]	50%	42.9%	7.1%
Das et al. [34]	36%	36%	28%
Present study	71.6%	15%	13.3%

Contrary to our findings, in a retrospective study of 906 individuals of COM by Yorgancilar et al., 511 (56.4%) had cholesteatoma, and 395 (43.6%) had granulation and/or polyp tissue [35]. Gupta et al. reported that the incidence of cholesteatoma was around three times that of both granulations and cholesteatoma & granulations combined [36].

## Conclusions

This review and the results emerging therein demonstrated that chronic inflammation of the middle ear in children suppresses the development of pneumatization in the temporal bone. The volume of the middle ear is not large enough to cause a retraction pocket and hence we believe that antrum size and status are also important in the formation of a COM-like retraction pocket in inactive squamosal disease due to its ability to exert a greater negative middle ear pressure.

However, a variety of vectors like population size, geographical background, genetic, racial and hereditary factors may influence varying cellularity of mastoid and by implication type and degree of changes in middle ear cleft along with the outcome of any therapeutic intervention. These factors also explain the divergence in the observations of various studies.
